# Antioxidant Capacity and Hepatoprotective Role of Chitosan-Stabilized Selenium Nanoparticles in Concanavalin A-Induced Liver Injury in Mice

**DOI:** 10.3390/nu12030857

**Published:** 2020-03-23

**Authors:** Kaikai Bai, Bihong Hong, Jianlin He, Wenwen Huang

**Affiliations:** 1 Third Institute of Oceanography, Ministry of Natural Resources, Xiamen 361005, China; bhhong@tio.org.cn (B.H.); hejianlin@tio.org.cn (J.H.); wwhuang@tio.org.cn (W.H.); 2 Technology Innovation Center for Exploitation of Marine Biological Resources, Ministry of Natural Resources, Xiamen 361005, China

**Keywords:** selenium nanoparticles, liver injury, antioxidant, concanavalin A

## Abstract

Selenium nanoparticles (SeNPs) have attracted wide attention for their use in nutritional supplements and nanomedicine applications. However, their potential to protect against autoimmune hepatitis has not been fully investigated, and the role of their antioxidant capacity in hepatoprotection is uncertain. In this study, chitosan-stabilized SeNPs (CS-SeNPs) were prepared by means of rapid ultra-filtration, and then their antioxidant ability and free-radical scavenging capacity were evaluated. The hepatoprotective potential of a spray-dried CS-SeNPs powder against autoimmune liver disease was also studied in the concanavalin A (Con A)-induced liver injury mouse model. CS-SeNPs with size of around 60 nm exhibited acceptable oxygen radical absorbance capacity and were able to scavenge DPPH, superoxide anion, and hydroxyl radicals. The CS-SeNPs powder alleviated Con A-caused hepatocyte necrosis and reduced the elevated levels of serum alanine transaminase, aspartate transaminase, and lactic dehydrogenase in Con A-treated mice. These results suggest that the CS-SeNPs powder protected the mice from Con-A-induced oxidative stress in the liver by retarding lipid oxidation and by boosting the activities of superoxide dismutase, glutathione peroxidase, and catalase, partly because of its ability to improve Se retention. In conclusion, SeNPs present potent hepatoprotective potential against Con A-induced liver damage by enhancing the redox state in the liver; therefore, they deserve further development.

## 1. Introduction

Selenium (Se) is an indispensable trace element required for animals and humans. It is a dietary nutrient playing an important role in many aspects of health [1,2], in particular for its antioxidant activity [1,2,3]. It is an integral element of the catalytic site of more than 25 selenoproteins and enzymes in the human body, and it plays an essential role in protecting cells and tissues from oxidative injury [2,3]. Se deficiency might break the balance between antioxidants and oxidants in the body, which can increase oxidation-associated risks, especially when the body is challenged by oxidative stress [1,2,3]. Generally, individuals do not require Se supplementation if a normal diet containing adequate Se is available, while people living in regions of low environmental selenium level (e.g., mainland China and Egypt) may be suffering from insufficient Se intake due to the low Se content in their food. It is necessary to meet the daily requirement of this nutrient by Se supplementation, especially for those subjected to Se deficiency [1,2,3,4].

Se deficiency is associated with numerous diseases including liver damage. Oxidative stress caused by various stimuli is an important aspect of many liver diseases elicited by toxic chemicals [5], autoimmune response [5,6], anoxic/reoxygenation injury [6], viral infection [5,6], alcoholic injury [6,7], and so on. Recently, an increasing number of studies have indicated that liver injury can be induced by Se deficiency, and Se supplementation is able to recover the hepatic damage [8,9,10,11,12]. More importantly, some hepatic diseases caused by alcohol, toxic elements, heavy metals, and chemotherapeutic drugs can be attenuated by extra Se supplementation [8,9,10,11,12]. It has been reported that Se might also help treat individuals with liver diseases such as hepatocellular carcinoma [13,14]. The antioxidant potential of Se plays an important part in the hepatoprotective role of this element.

Recently, selenium nanoparticles (SeNPs)—elemental, nanosized selenium particles—have attracted increasing attention due to their excellent properties and favorable biological activities [3,15,16]. SeNPs are considered to be a prospective Se supplement, since they have exhibited great potential for use in nutritional supplementation, chemoprevention, chemical therapy, and nanomedicine delivery applications [3,15,16]. The hepatoprotective effect of SeNPs seems to be promising, as SeNPs can protect animals from chemicals- or pathogen-induced hepatic damage through their antioxidant capacity [17,18,19,20]. However, the hepatoprotective effect of the nanoparticles has not been fully studied, especially when liver injury is caused by other factors such as an autoimmune disorder. In fact, whether the antioxidant activities of SeNPs may also protect from immune disorder-caused liver injury is uncertain.

Chitosan (CS), the only positively charged polysaccharide in nature, has been extensively explored for its potential use in drug delivery systems [21]. In this study, chitosan-stabilized selenium nanoparticles, namely CS-SeNPs, were prepared by means of rapid ultra-filtration (UF), and then the antioxidant activity and free-radical scavenging capacity of CS-SeNPs were evaluated. Furthermore, the hepatoprotective effect of dried CS-SeNPs powder against autoimmune liver disease was studied by using mice with concanavalin A (Con A)-induced liver injury, which are a model of this disease.

## 2. Materials and Methods

### 2.1. Materials and Animals

#### 2.1.1. Materials

CS of food grade (90.32% deacetylated, average molecular weight of 37 kDa) was provided by Aoxin Pharmaceutical Co. Ltd. (Taizhou, China). The compounds 2,2′-azobis-(2-amidinopropane)-dihydrochloride (AAPH), 1,1-diphenyl-2-picrylhydrazyl (DPPH), fluorescein disodium, pyrogallic acid, and 6-hydroxy-2,5,7,8-tetramethylchroman-2-carboxylic acid (Trolox) of high purity were obtained from Sigma-Aldrich (St.Lousi, MO, USA). Glycyrrhizic acid (GA) was purchased from Xi’an Liqun Pharmaceutical Co. Ltd. (Xi’an, China). Ascorbic acid (VC), sodium selenite, acetic acid, and other reagents of analytical grade were obtained from local commercial suppliers (Sinopharm Chemical Reagent Co., Ltd., Shanghai, China, etc). The assay kits for measuring protein content, alanine transaminase (ALT), aspartate transaminase (AST), lactic dehydrogenase (LDH), glutathione (GSH), thiobarbituric acid-reactive substances (TBARS), superoxide dismutase (SOD), catalase (CAT), and glutathione peroxidase (GSH-Px) were supplied by Jiancheng Bioengineering Institute (Nanjing, China).

#### 2.1.2. Animals

Male Kunming (KM) mice (8–10 weeks old, 18–22 g body weight) of specific-pathogen-free (SPF) grade were provided by Laboratory Animal Center, Shenyang Pharmaceutical University (Shenyang, China) with the license No. SCXK (Liaoning) 2015-0001. Mice were housed in a standardized sterile animal room with controlled temperature (25 ± 2 °C) and humidity (50% ± 10%) and light/dark cycle (12 h/12 h). The procedures utilized in the animal experiment were approved by the Animal Ethics Committees at Shenyang Pharmaceutical University (ethical committee approval number: SYPU-IACUC-C2017-5-17-104, Date (17/5/2017)). They were also compliant with the general recommendations and provisions of the Chinese Experimental Animals Administration Legislation.

### 2.2. Preparation, Characterization, and Stabilization of SeNPs

CS-SeNPs were synthesized in the presence of CS, as described in previous studies [15,16]. A CS-SeNPs colloid was obtained with the final concentrations of Se, CS, and VC of 4 mM, 0.1% (*w*/*w*), and 16 mM, respectively. The colloid was purified through UF in a 0.5% (w/w) acetic acid solution, unless the permeate did not fade the color of a 1 µmol/L potassium permanganate (KMnO_4_) solution within 20 min. A membrane filtration device (FlowMem-0015, Starmem Scitechnology, Xiamen, China) equipped with a polyethersulfone (PES) membrane (molecular weight cutoff (MWCO) = 8 kDa, UE008, GE, USA) was utilized to perform UF. After that, a purified CS-SeNPs colloid was obtained, and the content of CS-SeNPs was measured by lyophilizing the colloid. The size distribution, morphological characteristics, and zeta-potential of SeNPs were also investigated as previously described [15,16]. In addition, part of the purified CS-SeNPs were added to another stock of acetic acid solution containing CS, and then the mixture was spray-dried to acquire a dried CS-SeNPs powder, as reported in our previous studies [15,16]. Se content was determined by using inductively coupled plasma mass spectrometry (ICP-MS) [22].

### 2.3. Oxygen Radical Absorbance Capacity (ORAC) Test

The ORAC assay was performed by using fluorescein disodium as the probe molecule according to López-Alarcón [23,24], with some modification. Briefly, stock solutions of fluorescein disodium (50 μM) and AAPH (30 mM) were prepared freshly in deionized water. Reaction mixtures containing AAPH (20 μL) and fluorescein salt (20 μL), with or without the tested samples, were incubated in phosphate-buffered solution (PBS, 50 mM, pH 7.4) at 37 °C. The consumption of fluorescein, dependent on its incubation with AAPH, was evaluated from fluorescence measurements. The fluorescence measurements were conducted by utilizing a microplate reader (SpectraMax M5, Molecular Devices, CA, USA) equipped with a Softmax Pro software program, and the progressive decrease of fluorescence intensity (excitation: 485 nm; emission: 538 nm) was recorded. The ratio of current fluorescence (F) to initial fluorescence (F_0_), indicated as F/F_0_, was plotted as a function of time. The area under the curve (AUC) was integrated up to the time necessary for F/F_0_ to reach the value of 0.005. Each area was employed to obtain the relative net AUC value as follows:

Relative Net AUC = (AUC_sample_ − AUC_AAPH+_)/AUC_AAPH+_ (1)

where AUC_sample_ is the AUC of a sample, and AUC_AAPH+_ is the AUC of the AAPH control without any sample.

After that, the relative net AUC was plotted as a function of the concentration of the sample, and the slope value (SV) of each AUC curve was measured through linear regression. The ORAC of each sample was calculated as follows:

ORAC = SV_sample_/SV_Trolox_ (2)

where SV_Trolox_ is the slope value of the AUC for Trolox; SV_sample_ is the slope value of the AUC for the examined sample.

### 2.4. Free-Radical Scavenging Tests

The DPPH radical (•DPPH) and superoxide anion radical (•O_2_^−^) scavenging assays were performed as previously described [25] to measure the antioxidant abilities of the tested samples. In addition, the hydroxyl radical (•OH) scavenging activity was determined according to Peralta [26], with some modification. Fenton reagent (ferric sulphate/hydrogen peroxide, FeSO_4_/H_2_O_2_) was used to generate •OH, while salicylic acid (SA) was utilized as an •OH-trapping reagent to absorb the generated •OH. In brief, to a 1 M acetic acid/sodium acetate solution (pH 3.5) the tested sample was added at various concentrations, followed by the addition of Fenton reagent and SA. The mixture was quickly adjusted to 10 mL with ethanol, reaching final concentrations of FeSO_4_, H_2_O_2_, and SA of 0.36 mM, 0.1 mM, and 0.54 mM, respectively. After incubation for 30 min at 25 °C, the absorbance of the solution was recorded at 520 nm. The •OH scavenging ability was calculated as follows:

Scavenging ability (•OH) = (A_1_ − A_2_ + A_3_)/A_1_ × 100% (3)

where A_1_ is the absorbance of the control solution containing Fenton reagent and SA; A_2_ is the absorbance of the mixture of sample and reagents; A_3_ is the absorbance of the sample without any reagent.

### 2.5. Animal Experiments

#### 2.5.1. Experiment Design and Animal Grouping

A Con A challenge test was conducted as described by Zhang [27] and Anraku [28], with some changes. Briefly, 80 male KM mice were randomly divided into 8 groups (10 mice each): Control, Model, VC, GA, CS, and three CS-SeNPs groups (L-Se, M-Se, and H-Se). The mice were daily administered the above treatments by gavage for 35 days (shown in Table 1), and their body weight (bw) was monitored throughout the experiment. Except for the normal control, mice were also intravenously (iv) administrated Con A (20 mg/kg bw) in their tail veins after the last pre-treatment. The mice in the control group were given the same volume of sterile normal saline instead of Con A. The mice were allowed free access to water. Eight hours later, the mice were anesthetized through intraperitoneal (ip) injection of aqueous pentobarbital sodium (60 mg/kg bw). The blood of the animals was collected into heparin-free tubes to acquire the sera, while tissue samples were also obtained.

#### 2.5.2. Liver Histopathology Assessment

Fresh tissue obtained in the middle of the hepatic left lateral lobe was preserved in 4% paraformaldehyde for at least 24 h. After that, the specimen was embedded in paraffin and then sectioned at a thickness of ~5 μm for hematoxylin and eosin (H&E) dual staining. The tissue damage was observed by light microscopy and was graded by an independent viewer blinded to the treatment, as described by Wu [29] with slight modifications. Briefly, hepatic injury was scored on the basis of the severity and the area of necrosis as follows: 0 (normal); 1 (rare necrosis); 2 (necrosis <25%), 3 (25% ≤ necrosis < 50%), 4 (50% ≤ necrosis < 75%) and 5 (75%≤ necrosis).

#### 2.5.3. Determination of Se Deposition 

Se in the liver blood was determined by ICP-MS assay, as previously described [15,16]. Briefly, the sample was mixed with HNO_3_ and H_2_O_2_, and the mixture was digested in a microwave device (JK-MDA-23, Shanghai, China). After digestion, the Se content in the digested solution was determined by ICP-MS (7700X, Agilent, CA, USA).

#### 2.5.4. Biochemical Analysis

Fresh liver samples were rinsed with cold normal saline and then homogenized in ice-cold saline. After immediate centrifugation (10,000× *g*, 4 °C, 10 min), the supernatants were collected. After that, the levels of AST, ALT, LDH, TBARS (malondialdehyde equivalent), GSH, GSH-Px, SOD, and CAT, were measured in the liver supernatants and sera, following the instructions of the commercial kits.

### 2.6. Statistical Analysis

For all experiments, data are presented as mean ± standard deviation (SD). The difference between three or more groups was analyzed through the one-way analysis of variance (one-way ANOVA) test followed by multiple comparisons. SPSS software program (version 17.0 for Windows, Microsoft, Redmond, WA, USA) was used for data processing, and a *p* value of < 0.05 was regarded as statistically significant.

## 3. Results and Discussion

### 3.1. UF-assisted Preparation of CS-SeNPs

Many polysaccharides, such as chitosan, alginate, gum arabic, and sialic acid, have been used to synthesize and stabilize SeNPs, and SeNPs of various shapes have been obtained [15,16,30,31,32,33]. These polysaccharides are able to control the synthesis, morphological characteristics, and structure of SeNPs and might also stabilize the nanoparticles. However, it is challenging to remove the synthesis byproducts from SeNPs. In some previous reports, a washing process by high-speed centrifugation was repeated several times to eliminate the unwanted impurity [34,35,36]. In other studies, dialysis was utilized to purify the synthesized SeNPs, followed by a lyophilization process to obtain the final SeNPs product [15,16,30,32,33]. However, these methods are impracticable for industrial production due to their low efficiency, limited production scale, and high cost. Besides, the stability of SeNPs should be taken into account during the purification process.

**Figure 1:**
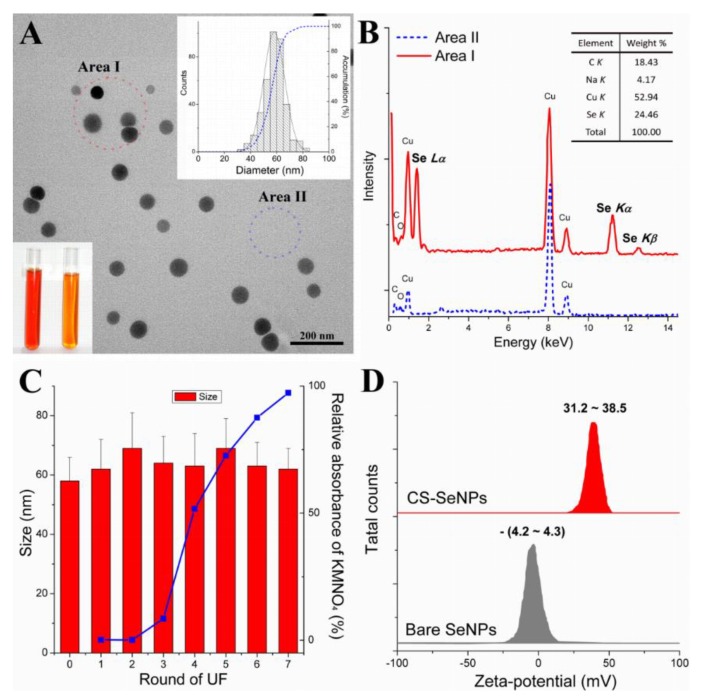
Physicochemical characteristics of CS-SeNPs. (**A**) TEM image, appearance, and size distribution of CS-SeNPs. (**B**) Typical EDS spectra of CS-SeNPs (measured at Area I, Figure 1A) and elemental composition (inset). (**C**) Size of SeNPs after rounds of ultra-filtration (UF) and absorbance (measured at 525 nm) of a KMnO_4_ solution mixed with corresponding UF permeates. (**D**) Zeta-potentials of CS-SeNPs (UF Round 0–6) and of bare SeNPs.

Herein, UF was utilized, instead of dialysis or centrifugation, to purify CS-SeNPs. The UF membrane used in this study allowed free access to soluble compounds with small molecular weight (e.g, VC and its oxides), which could induce fading of the color of the KMnO_4_ solution but entrapped CS and SeNPs. After the UF process, monodisperse CS-SeNPs were obtained with an average size of around 60 nm, as shown in Figure 1A. Some characteristic peaks (1.37, 11.22, and 12.49 keV) presented in the EDS spectra of SeNPs were identified as Se *L_α_*, Se *K_α_*, and Se *K_β_* signals, respectively, confirming the elemental nature of these nanoparticles (Figure 1B). More importantly, SeNPs were stable when the soluble byproducts were removed by UF, as evidenced by the stable size of SeNPs and the decreasing ability of UF to induce fading of the KMnO_4_ solution (Figure 1C). This was associated with a high zeta-potential of CS-SeNPs (+31.2 ~ +38.5 mV), as compared with that of bare SeNPs (−4.3 ~ −4.2 mV) (Figure 1D). Evidently, the surface decoration by CS significantly elevated the zeta-potential of SeNPs, which might be partly attributed to the positively charged −NH^3+^ groups in CS [15,16,33]. This might guarantee the stability of SeNPs throughout the UF process. In short, UF is usually faster than dialysis and might retain the shape, size, and structure of SeNPs whiole removing byproducts. It might allow a rapid preparation of SeNPs.

### 3.2. Antioxidant Capacity of CS-SeNPs Determined by the ORAC Methodology

**Figure 2:**
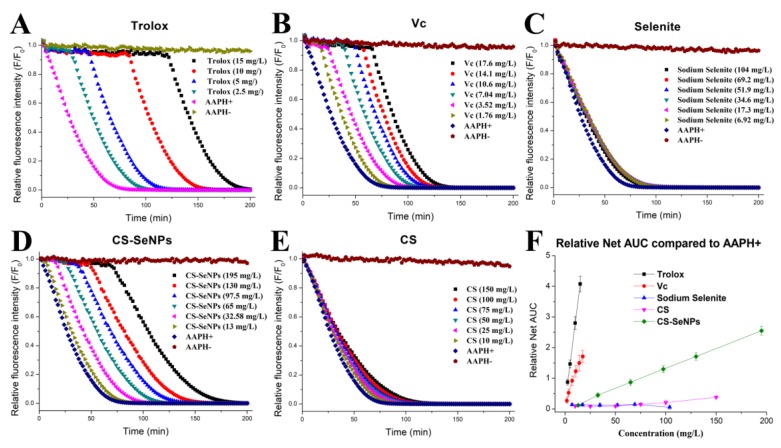
Effect of (**A**) Trolox, (**B**) ascorbic acid (VC), (**C**) sodium selenite, (**D**) CS-SeNPs colloid, and (**E**) CS on fluorescein consumption induced by AAPH and (**F**) dependence of the relative net area under the curve (AUC) upon the concentration of the compounds examined.

The ORAC assay [23,24] was performed to estimate the total antioxidant potential of the Se samples. Trolox and V were used as referenced antioxidant compounds. As shown in Figure 2, the fluorescence was stable in the dark for 200 min without AAPH (indicated as AAPH-) but it was strongly weakened by the addition of AAPH (indicated as AAPH+). The fluorescence decay induced by AAPH, however, could be delayed by antioxidants. Trolox, VC, and CS-SeNPs, as presented in Figure 2, were able to hinder the APPH-induced fluorescence decay in a dose-dependent manner, whereas sodium selenite (6.92–104 mg/L) showed little inhibition due to its high chemical valence (Figure 2C).

To evaluate the antioxidant ability of the tested samples, the relative net AUC of each sample was plotted as a function of concentration (Figure 2F), followed by the calculation of the ORAC results, as shown in Table 2. It was found that the antioxidant abilities of the tested samples varied. According to the results of the ORAC assay, the antioxidant ability of the samples was: Trolox > VC > CS-SeNPs > CS > selenite, consistent with the order of chemical valence of Se. The ORAC of CS-SeNPs was around 1/7 of that of VC and approximately 1/20 of that of Trolox. This indicates an acceptable antioxidant capacity of the SeNPs colloid. In the colloid, the population of SeNPs was small, as only about 20% of the weight of the lyophilized CS-SeNPs could be identified as Se (measured by ICP-MS [20]). However, the ORAC of CS-SeNPs was much greater than that of CS (Table 2). SeNPs might significantly improve the ORAC of CS-SeNPs, though the ORAC of these nanoparticles by themselves was difficult to measure, due to their very limited stability [15,16].

### 3.3. Free-Radical Scavenging Ability of CS-SeNPs

Many liver diseases are accompanied by an excess of radical oxygen species (ROS), which can result in oxidative damage to proteins and lipids in the body. Some saccharide- or protein-decorated SeNPs were reported to be able to clear free radicals, though in these studies, the stabilizer–SeNPs were regarded as a whole when evaluating their radical-scavenging potential [33,37,38]. However, the free-radical scavenging abilities of the stabilizers should be taken into consideration when measuring this property for SeNPs combined with a stabilizer. Besides, other reference compounds need to be studied at the same time, as they could help to evaluate the potential of SeNPs in clearing free radicals. Nonetheless this was not done in many studies [30,33,37,38].

**Figure 3:**
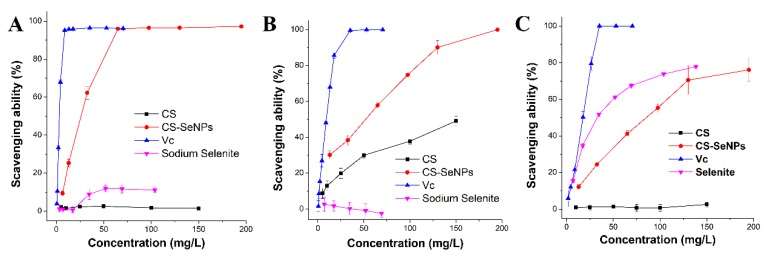
Free-radical scavenging activities against (**A**) DPPH (•DPPH), (**B**) superoxide anion (•O_2_^-^), and (**C**) hydroxyl (•OH^-^) radicals.

Herein, •DPPH, •O_2_^-^ and •OH^-^ were utilized as model free radicals to evaluate the radical scavenging activities of CS-SeNPs, CS, sodium selenite, and VC. As shown in Figure 3, the CS-SeNPs colloid was able to remove all the three free radicals in a dose-dependent fashion, with scavenging ability of 1/7 (•DPPH), 1/3 (•O_2_^-^), and 1/5 (•OH^-^) of that of VC. CS-SeNPs presented a stronger activity than sodium selenite in clearing •DPPH and •O_2_^-^, while they were not superior to selenite in scavenging •OH^-^ (Figure 3 and Table 3). This suggests that the potential of CS-SeNPs in reducing free radicals is complex. In addition, great differences in radical scavenging capacities could be found between CS and CS-SeNPs (Table 3). SeNPs might greatly contribute to the radical scavenging capacity of CS-SeNPs, when considering the weak potential of CS in cleaning these free radicals. This could be partly attributed to the low chemical valence and the vast specific surface area of these nanoparticles.

### 3.4. Hepatoprotection by Dried CS-SeNPs against Con A

#### 3.4.1. Dried CS-SeNPs Powder Used as a SeNPs Sample

The CS-SeNPs colloid is unavailable for commercial application in oral administration systems due to its low stability [15,16]. In some previous studies [15,16], the CS-SeNPs colloid alone or mixed with additional CS solution, was spray-dried to prepare a dried CS-SeNPs powder for better storage stability and larger application in the clinic. In this study, UF-purified CS-SeNPs were used to prepare a dried CS-SeNPs powder sample containing 30 mg/kg of Se (measured by ICP-MS [22]). The dried CS-SeNPs powder was utilized to evaluate the protective potential of SeNPs against Con-A-induced liver injury.

Oral administration of CS-SeNPs powder (0.6, 1.2, 3.6 mg/kg bw, each day) to mice for 35 days was used to simulate sub-chronic Se consumption by humans. These doses were equal to 18, 36, and 108 μg (Se)/kg bw in terms of Se dose, respectively, according to the equivalent dose conversion of mice to human (≈ 12:1) based on body surface area [25,39]. Considering the body weight of a human adult (generally 60 kg), the daily Se doses (1.5, 3, and 9 μg Se/kg bw) for humans could actually be 90, 180, and 540 μg Se per day, which meets the human requirements of adequate or super-nutritional Se [1,2,3].

#### 3.4.2. Growth and Viscera Status of KM Mice

**Figure 4:**
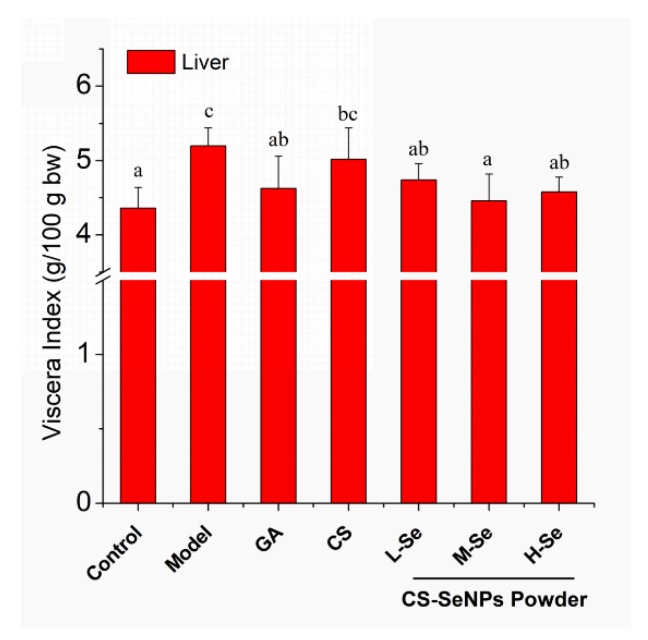
Liver index of KM mice, defined as the ratio of live weight to body weight. ^a–c^ Means within a panel with different letters differ significantly.

The biosafety of SeNPs should be taken into account before exploring their potential in the clinic. As previously reported, SeNPs were much safer when compared with selenite [2,15,16]. Herein, mice were given dried CS-SeNPs at three doses (0.6, 1.2, 3.6 mg/kg bw) well below the LD_50_ [15,16]. The body weight of the mice was recorded to monitor their growth, showing that the growth was comparable among the animals throughout the experiment. Besides, the viscera indexes of heart, spleen, kidney, and thymus were normal among all groups of mice, except for that of liver. Con-A injection led to hepatic edema, characterized by a significantly higher liver index (*p* < 0.05, Model versus Control), as presented in Figure 4. However, this could be prevented by the oral administration of GA or dried CS-SeNPs powder, indicating the hepatoprotective potential of CS-SeNPs.

#### 3.4.3. Histological Assessment of Hepatic Damage

**Figure 5:**
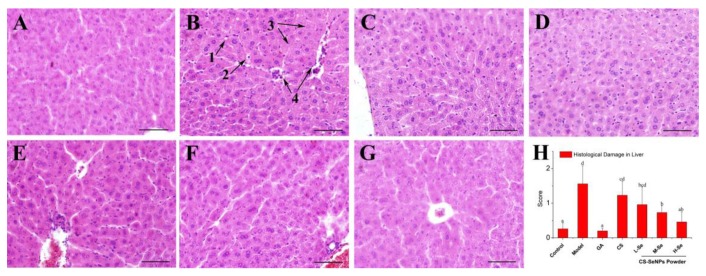
Histology of liver sections upon hematoxylin and eosin (H&E) staining 200×. (**A**) Normal control, (**B**) Model, (**C**) GA, (**D**) CS, (**E**) L-Se, (**F**) M-Se, (**G**) H-Se, and (**H**) histological damage score of each group. Scale bars represents 25 μm. Arrows: 1, ballooning degeneration; 2, hydropic degeneration; 3, lytic necrosis; 4, inflammatory cell infiltration. ^a–d^ Means within a panel with different letters differ significantly.

To evaluate the protective activity of the Se supplement against Con-A-caused liver injury, mice liver sections were observed by microscopy after H&E staining. As illustrated in Figure 5B, severe hepatic damage, including ballooning degeneration, hydropic degeneration, pyknotic nuclei, lytic necrosis, and inflammatory cell infiltration, was observed 8 h after Con-A-injection, as compared with the Control (Figure 5A), consistent with previous studies [27,28]. However, the hepatic injury could be relieved by the oral administration of GA or dried CS-SeNPs, as evidenced by the amelioration of the aforementioned injuries, especially cellular necrosis (Figure 5C–G). Additionally, the lesions were scored blindly by a pathologist to grade the liver injury, as described in the Materials and Methods section. The results, presented in Figure 5H, indicated that CS-SeNPs were able to protect the mice from Con-A-induced liver injury in a dose-dependent manner. The significant difference between the CS group and the M-Se group (*p* < 0.05) suggested an essential contribution of SeNPs to liver protection by dried CS-SeNPs.

#### 3.4.4. Se Retention

**Figure 6:**
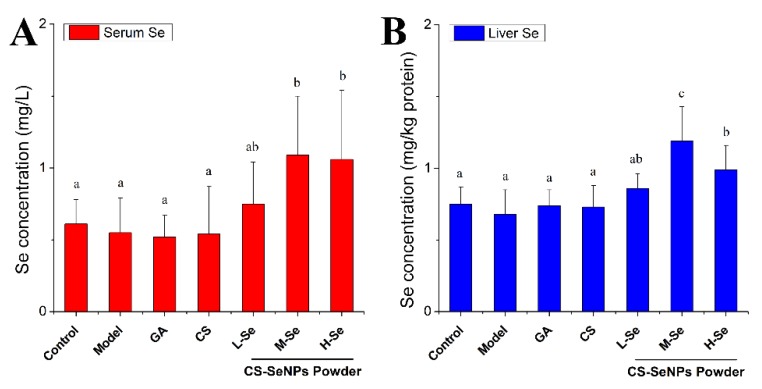
(**A**) Serum Se concentration and (**B**) hepatic Se level in KM mice. The administration modalities in each group are shown in Table 1. ^a–c^ Means within a panel with different letters differed significantly (*p* < 0.05).

Se deposition in mice bodies was monitored to study the Se-supplying ability of CS-SeNPs in mice. This might help to investigate the role of SeNPs within the CS-SeNPs powder in protecting the liver from Con-A-induced damage. As presented in Figure 6, the serum and hepatic Se levels in the Se-supplementation groups were significantly higher than those of other groups (*p* < 0.05), indicating that CS-SeNPs might enhance the Se stock in the animals. This is in line with the reported efficient Se-retention capacity of some SeNPs stabilized by other macro-molecules [2,3,25,40] or microorganisms [17], confirming the potential of nanoparticles in Se supplementation.

Se retention by extra Se supplementation needs to be discussed. One issue was the Se source. In consideration of the daily intake of adult KM mice (4–8 g diet/day each) [16,41] and the extremely low Se content of the feed (<0.1 μg Se/g diet) in this study, CS-SeNPs were actually the main source of Se. In addition, a great difference in Se levels was found between the M-Se group and the CS group (*p* < 0.05). It The SeNPs within the CS-SeNPs powder contributed to the enhancement of Se retention. Another issue was the dose of SeNPs. The daily doses of SeNPs used in this hepatoprotection study were acceptable, as they were far from those inducing a toxic status and were able to fulfill the need of adequate or super-nutritional Se according to Barnes [42] and Raines [43].

#### 3.4.5. Serum Levels of Liver Enzymes and GSH

Serum enzymes, such as LDH, ALT, and AST, are generally recognized as essential bio-chemical markers of liver injury. They will present increased activity when hepatocytes are necrotic, due to their large leakage from the liver into the blood stream [44]. As presented in Table 4, serum ALT, AST, and LDH levels increased 8 h after intravenous Con A administration compared with the normal control group (*p* < 0.05), suggesting hepatocellular damage caused by Con A. However, pretreatment with GA (10 mg/kg bw) or dried CS-SeNPs (0.6, 1.2, 3.6 mg/kg bw) was able to protect the mice from Con-A-induced liver injury, as indicated by a significant decrease in these enzymes’ levels as compared with the Model group (*p* < 0.05). Besides, the serum GSH concentration was also investigated, since a dramatic increase of these enzymes is often found in the damaged liver in the presence of oxidative stress [18,29,45]. The data shown in Table 4 indicate that CS-SeNPs (1.2 or 3.6 mg/kg bw) significantly attenuated Con-A-induced reduction of serum GSH (*p* < 0.05, versus Model). Possibly the antioxidant activity of CS-SeNPs contributes to hepatoprotection.

Great difference in the levels of the aforementioned enzymes could be observed when comparing the M-Se group with the Control group (*p* < 0.05, shown in Table 4), which attracted our attention. It suggested SeNPs play an essential role in protecting hepatocytes from liver necrosis caused by Con A. Similar superiority of CS-SeNPs to CS was also found when studying serum GSH levels. These results are in good agreement with the observed histopathological changes (Figure 4 and Figure 5) and Se retention modification (Figure 6), suggesting that the hepatoprotection function of the CS-SeNPs powder may be greatly attributed to SeNPs.

#### 3.4.6. Antioxidant Activity in the Liver

Oxidative stress is very common in hepatitis. It is often accompanied by the development of inflammatory liver injury induced by autoimmunity disorders [29,46]. In Con-A-induced acute liver injury, T and NKT cells are activated by Con A, producing inflammatory cytokines and chemokines [29,46,47]. These factors recruit and activate more immune cells which would attack hepatocytes, leading to severe necrosis [46,47]. Throughout the course of this process, oxidative burst occurs quickly and involves a rapid jump of ROS production, resulting in the oxidation of a diverse range of biological molecules, such as nucleic acids, lipids, and proteins [27,29]. The alleviation of the oxidative risk might be helpful for both ameliorating the oxidation-induced damage in cells and controlling the activation of critical signaling cascades such as protein kinases, cytokines, and transcription factors [6,27], finally protecting against hepatic necrosis and dysfunction.

The enhancement of serum GSH level by CS-SeNPs (shown in Table 4) indicated the possible antioxidant activity of the Se-supplement in animals. Herein, the antioxidant activity of CS-SeNPs in the liver was also studied. As shown in Table 5, Con A injection led to a potent oxidative stress characterized by an increase of TBARS and a decrease in GSH. It also led to the downregulation of some vital antioxidant enzymes including SOD, CAT, and GSH-Px. Apparently, the hepatic cells suffered from severe oxidative stress after the injection of Con A. However, the CS-SeNPs powder at the studied doses maintained GSH levels, reduced TBARS levels, and upregulated the levels of SOD, CAT, and GSH-Px, when compared with the Model group (*p* < 0.05). This powder (1.2 and 3.6 mg/kg bw) showed powerful antioxidant ability, comparable or superior to that of GA (10 mg/kg bw). Its hepatoprotection mechanism might be different from that of some primary medicines such as cortisone (a hormone inhibiting the inflammatory process), while Se immunological modulation ability [1,2,3] could also take part in the protection. This deserves further investigation.

Some points needed to be discussed. The first regards the contributions of SeNPs and CS to the overall antioxidant ability of CS-SeNPs. CS (1.2 mg/kg bw) did not improve the above-mentioned biomarkers, but dried CS-SeNPs powder containing an almost equal amount of CS was able to enhance the antioxidant status in mice, as shown by the decrease in TBARS and the upregulation of both SOD and GSH-Px (*p* < 0.05 versus Model). It is probable that SeNPs made a great contribution to the antioxidant activity of the CS-SeNPs powder in animals. The second point is the role of SeNPs in improving the antioxidant state in vivo. Se deposition (shown in Figure 6) and the intrinsic antioxidant potentials of SeNPs (presented in Table 2 and Table 3) might partly explain the antioxidant ability of SeNPs in the liver, though the Se concentration achieved might have been too low to remove the oxidative stress directly. More importantly, SeNPs were efficient in boosting GSH-Px, which is a vital antioxidant enzyme that uses Se as an integral element of its catalytic sites. It is possible that Se retention by CS-SeNPs contributed greatly to the Se storage pool in the body [48], finally resulting in an enhancement of GSH-Px. The third point is the broad hepatoprotection of SeNPs. It was reported that SeNPs alone or combined with vitamins might protect animals from the hepatic injury caused by toxic chemicals (e.g, carbon tetrachloride [17], acetaminophen [18], and acrylamide [19]) or by pathogen infections (e.g, *Schistosoma* spp. [20]). These reports focused on the potential of SeNPs to enhance the redox state in vivo and showed that it really contributed to the hepatoprotection of SeNPs. In the present study, CS-SeNPs were able to protect mice from Con-A-induced autoimmune hepatitis by improving the redox status and enhancing the levels of SOD, CAT, and GSH-Px. SeNPs might play a passive role in protecting animals from liver diseases involving serious oxidative stress.

## 4. Conclusions

In this study, a rapid and simple method involving UF was introduced to prepare CS-SeNPs of around 60 nm. The CS-stabilized SeNPs exhibited acceptable antioxidant activity and potent free-radical scavenging ability. A spray-dried CS-SeNPs powder was able to protect mice from Con-A-induced autoimmune liver injury, as indicated by the reduction of hepatic edema, amelioration of hepatocytes necrosis, and alleviation of the leakage of ALT, AST, and LDH from the hepatocytes into the blood stream. The hepatoprotective capacity of the dried CS-SeNPs powder might be partly attributed to the ability of this Se-supplement to improve Se retention, reducing TBARS, increasing GSH, and up-regulating SOD, CAT, and GSH-Px in the liver. SeNPs might contribute greatly to the antioxidant activity and hepatoprotective role of the CS-SeNPs. In summary, SeNPs stabilized by CS deserve being considered for further development as nutrient supplements or even nanomedicines that aim to defend against autoimmune-disorder-induced hepatic injury.

## Figures and Tables

**Table 1:** Administration of the treatments during the concanavalin A (Con A) challenge experiment (Kunming (KM) mice, *n* = 10).

Group	Pre-treatment (once daily, ig, Day 1–35)	Treatment (once, iv, Day 35)
Control	Normal saline	Normal saline
Model	Normal saline	Con A (10 mg/kg bw)
GA	Glycyrrhizic acid (10 mg/kg bw)	Con A (10 mg/kg bw)
CS	Chitosan (1.2 mg kg−1 bw)	Con A (10 mg/kg bw)
L-Se	Dried CS-SeNPs power (0.6 mg kg−1 bw)	Con A (10 mg/kg bw)
M-Se	Dried CS-SeNPs power (1.2 mg kg−1 bw)	Con A (10 mg/kg bw)
H-Se	Dried CS-SeNPs power (2.4 mg kg−1 bw)	Con A (10 mg/kg bw)

Note: bw, body weight; ig, intragastrically; iv, intravenously; SeNPs; selenium nanoparticles

**Table 2:** Oxygen radical absorbance capacity (ORAC) results (*n* = 3).

Sample	ORAC ^1^ (mg Trolox/100 mg)	Relative ORAC ^2^ (%)
Trolox	100	100
VC	35.16 ± 3.54	35.16
Selenite	<0.001	<0.001
CS-SeNPs	5.10 ± 0.22	5.10
CS	0.75 ± 0.11	0.75

^1^ Values evaluated as described in the experimental section. Values represent the weight (mg) of Trolox that produces the same effect as 100 mg of a tested sample. ^2^ Values represent the ORAC of a sample compared with that of Trolox.

**Table 3:** EC_50_ of the free-radical scavenging ability (mg/L, *n* = 3) of the tested compounds.

Free Radical	VC	Sodium selenite	CS	CS-SeNPs
•DPPH	2.90 ± 0.04	>>150	>>150	20.2 ± 0.6
•O_2_^-^	5.79 ± 1.0	>>150	>150	17.5 ± 0.6
•OH^-^	15.0 ± 1.2	34.8 ± 1.3	>>150	75.1 ± 6.1

Note: EC_50_, concentration for 50% of maximal effect.

**Table 4:** Serum alanine transaminase (ALT), aspartate transaminase (AST), lactic dehydrogenase (LDH), and glutathione (GSH) levels in KM mice (*n* = 10).

Group	ALT(U/L)	AST(U/L)	LDH (U/L)	GSH (μmol/L)
Control	10.3 ± 2.1 ^ab^	13.2 ± 6.0 ^a^	2929 ± 396 ^a^	17.4 ± 7.8 ^a^
Model	32.1 ± 9.2 ^c^	62.3 ± 22.5 ^b^	3655 ± 331 ^c^	5.9 ± 4.9 ^b^
GA	15.7 ± 5.3 ^ab^	10.7 ± 6.0 ^a^	3151 ± 233 ^ab^	7.2 ± 5.4 ^b^
CS	20.3 ± 8.0 ^b^	54.2 ± 24.6 ^b^	3411 ± 185 ^bc^	7.1 ± 2.9 ^b^
L-Se	16.4 ± 6.1 ^ab^	26.1 ± 5.2 ^a^	2920 ± 349 ^a^	8.2 ± 3.3 ^b^
M-Se	7.1 ± 4.8 ^a^	13.9 ± 6.5 ^a^	2983 ± 277 ^a^	17.4 ± 8.4 ^a^
H-Se	20.2 ± 13.1 ^b^	60.6 ± 30.2 ^b^	3342 ± 517 ^abc^	21.7 ± 5.4 ^a^

^a–c^ Values within a column not sharing a common superscript letter differ significantly at *p* < 0.05.

**Table 5:** The levels of GSH, thiobarbituric acid-reactive substances (TBARS), superoxide dismutase (SOD), catalase (CAT), and glutathione peroxidase (GSH-Px) in mice liver (*n* = 10).

Group	GSH (μmol/g prot)	TBARS (μmol/g prot)	SOD (U/g prot)	CAT (U/g prot)	GSH-Px (U/g prot)
Control	7.99 ± 2.81 ^ab^	1.13 ± 0.35 ^a^	243 ± 39 ^a^	41.4 ± 7.5 ^ab^	473 ± 146 ^ab^
Model	2.08 ± 1,75 ^c^	4.68 ± 1.79 ^c^	154 ± 22 ^c^	28.9 ± 6.2 ^a^	183 ± 103 ^c^
GA	3.26 ± 2.01 ^bc^	1.28 ± 0.43 ^a^	151 ± 21 ^c^	56.6 ± 26.1 ^b^	399 ± 58 ^abc^
CS	4.69 ± 3.03 ^abc^	3.22 ± 1.06 ^bc^	184 ± 20 ^bc^	30.3 ± 10.9 ^a^	278 ± 133 ^bc^
L-SeM	2.37 ± 1.82 ^bc^	1.44 ± 0.50 ^ab^	nt	34.6 ± 13.2 ^ab^	450 ± 162 ^ab^
M-SeM	7.91 ± 3.81 ^ab^	0.96 ± 0.50 ^a^	240 ± 34 ^a^	50.4 ± 13.9 ^ab^	519 ± 97 ^a^
H-SeM	9.10 ± 3.56 ^a^	1.44 ± 1.06 ^ab^	204 ± 27 ^ab^	34.9 ± 7.7 ^ab^	461 ± 114 ^ab^

^a–c^ Values within a column not sharing a common superscript letter differed significantly at *p* < 0.05. prot, protein; nt, not tested.

## Abbreviations

AAPH	2,2’-Azobis-(2-amidinopropane)-dihydrochloride
ALT	alanine transaminase
AST	aspartate transaminase
AUC	area under the curve
bw	body weight
Con A	concanavalin A
CS	chitosan
CS-SeNPs	chitosan-stabilized selenium nanopartilcels
DPPH	1,1-diphenyl-2-picrylhydrazyl
•DPPH	1,1-diphenyl-2-picrylhydrazyl radical
EC_50_	concentration for 50% of maximal effect
EDS	energy-dispersive X-ray spectroscopy
GA	glycyrrhizic acid
GSH	glutathione
GSH-Px	glutathione peroxidase
ICP-MS	inductively coupled plasma mass spectrometry
i.p,	intraperitoneal
i.v,	intravenously
LDH	lactic dehydrogenase
KM	Kunming
KMnO_4_	potassium permanganate
LD_50_	median lethal dose
MW	molecular weight
MWCO	molecular weight cut-off
•O_2_^−^	superoxide anion radical
•OH^−^	hydroxyl radical
ORAC	oxygen radical absorbance capacity
RH	relative humidity
ROS	radical oxygen species
SA	salicylic acid
Se	selenium
SeNPs	selenium nanoparticles
SOD	superoxide dismutase
SPF	specifc-pathogen-free
TBARS	thiobarbituric acid-reactive substances
TEM	transmission electron microscopy
Trolox	6-hydroxy-2,5,7,8-tetramethylchroman-2-carboxylic acid
UF	ultra-filtration
Vc	ascorbic acid
